# Report of the Committee of Amer. Med. Ass’n, Etc.

**Published:** 1871-12

**Authors:** 


### SELECTIONS.

### REPORT OF THE COMMITTEE

*Appointed at the Annual Meeting of the American Medical Association, in New Orleans, in May, 1869, to Present Certain Resolutions, Adopted by the Association, to the Several State Medical Societies, and Ask Their Sanction of the Same. Read May 2, 1871.*

[From Transactions Amer. Med. Association, 1871.]

YOUR Committee ask leave respectfully to report as follows:—

Soon after the adjournment of the annual meeting of this Association, held in New Orleans, May, 1869, the Chairman of your Committee caused the preamble and resolutions referred to the several State Medical Societies to be printed in the form of a circular letter; and a copy of the same was sent to the proper officers of every State Medical Society, the existence of which, and the address of whose officers, could be ascertained. The resolutions and accompanying letter are as follows:

***Preamble and Resolutions adopted by the American Medical Association, in May, 1869.***

WHEREAS, The history of medical legislation in the various States of the Union clearly shows that no reliance can be placed on either the uniformity or the permanency of any laws relating to the practice of medicine; and

WHEREAS, The results of all the efforts made during the last twenty-five years, to elevate the standard of medical education, through concert of action among the numerous medical colleges of this country, have proved with equal clearness that such concert of action, in an efficient manner, is unattainable: therefore, be it

*Resolved*, That whatever is done to establish and maintain a just and fair standard of medical education throughout our whole country must be done by the profession itself, through its own voluntary organizations, in the same manner that it now establishes and enforces its code of ethics. The profession is as competent to declare, through its representatives in the National, State, and Local societies, what shall be the standard of attainments for those to be recognized and admitted into its ranks, and to establish the boards or agencies by which compliance with such standard shall be ascertained, as it is to declare what shall be the ethical rules governing the conduct of those already admitted.

*Resolved*, That this Association earnestly requests each State Medical Society to appoint annually one or more Boards of Examiners, composed of five thoroughly competent members, whose duty it shall be to meet, at suitable times and places, for the examination of all persons, whether graduates of colleges or not, who propose to enter upon the practice of medicine in their respective States, except such as have been previously examined and licensed by a similar Board, in some other State.

*Resolved*, That each State Medical Society be requested to make such regulations concerning the pay of the Board of Examiners, and the fee to be charged for a license to practise, that the former shall in no case depend on the amount received from the latter.

*Resolved*, That each State Medical Society be requested to require its Examining Board or Boards to exact of every applicant for examination adequate proof that he has a proper general education, is twenty-one years of age, and has pursued the study of medicine three full years, one-half of which time shall have been in some regularly organized medical college, whose curriculum embraces adequate facilities for didactic, demonstrative, and hospital clinical instruction.

*Resolved*, That each State Medical Society be requested to act on the foregoing propositions at the next regular annual meeting after the reception of copies of the same, and if approved and adopted by the State Medical Societies of two-thirds of the States, this Association shall deny representatives from all organizations who longer refuse to comply with the same, and shall recommend the State Societies to do the same; and all persons who after that date seek to enter upon the practice of medicine without first receiving a licence from the State Board of Examiners, shall be treated ethically as irregular practitioners.

*Resolved*, That in adopting the foregoing resolutions, by which it is proposed to treat the medical college diploma the same as the diploma of any literary college, this Association is actuated by no desire to injure the medical schools of our country. On the contrary, by the adoption of the fourth resolution, at the same time that the value of the mere college diploma is practically nullified, it is the desire, and confident expectation, that those institutions will be greatly benefited; because they will be forced to rival each other in the extent and efficiency of their courses of instruction, instead of the number of diplomas which they can annually distribute.

CHICAGO, July 1, 1869.

A. B. Secretary of ——.

DEAR SIR: Be pleased to present the accompanying preamble and resolutions to the first regular meeting of the ————, and ask for them a careful consideration. They were adopted with much unanimity by the American Medical Association, at its meeting in New Orleans in May last. The undersigned were appointed a committee with instructions to have them presented to all the State Medical Societies in our country. Please inform the Chairman of the Committee as early as possible, what action your society takes concerning them, and much oblige yours truly,

N. S. DAVIS, of Chicago, Ill.
J. M. TONER, of Washington, D. C.
J. S. WEATHERLY, of Montgomery, Ala.

A copy of the preamble and resolutions was also furnished to all the medical periodicals of our country.

The anniversary meetings of many of the State Medical Societies for 1869 had been held before the meeting of this Association in that year, consequently there occurred no opportunity to procure the action of such societies until 1870. In all of them, at least so far as ascertained by your Committee, the communication was respectfully received.

In a large majority of them it was referred to a special committee, with instruction to report at the next annual meeting. In the Medical Society of the State of New York, the communication was recieved, discussed, and laid on the table, without indicating any disposition to take further action concerning it.

In the Medical Society of the State of New Hampshire, the communication was laid on the table, and ordered printed. In the State Societies of Pennsylvania, Ohio, Indiana, Illinois, Michigan, Iowa, Missouri, Kentucky and Alabama, it was received and referred to special committees, whose reports have either not been made, or if made, have not been definitely acted upon by their respective societies. In the societies of Maryland, New Jersey, Wisconsin, and Kansas, the communication was received, discussed, and the several propositions fully approved. From the societies of the remaining States no answer to the communication has yet been received, by your Committee. It will be seen by the foregoing recital of facts, that the whole subject is still under consideration in the State Societies of a large majority of the States. And here it might be thought best to leave it, until a more complete expression had been received, were it not for the fact that, since the action of this Association, submitting the subject to the State Medical Societies, additional circumstances have been developed, having an important bearing in favor of some action by such societies. Although the second part of the preamble accompanying the resolutions, referred to the State Societies, assumed that efficient concert of action by the medical colleges of this country, was unattainable yet there was pending an unfinished movement, having for its sole object the procurement of such concert through the agency of a convention of delegates from the schools alone. One meeting had been held in Cincinnati, in May, 1867, which resulted in the harmonious adoption of a very desirable revision of the whole system of medical college instruction in our country. But so large a number of colleges were not represented that its action was made simply recommendatory, and a committee appointed to present the plan adopted to all the medical college faculties, and, if necessary, to call another convention. After the whole subject had been before the colleges for two years, the committee deemed it advisable to call a second convention, which was held in the city of Washington, in May, 1870. The representation present, however, was no more complete than in the first convention, and several of those who were present, representing some of the oldest and most influential colleges in the country, disclaimed having any authority to take such action as would be binding upon the institutions they represented,and hence the second convention ended in simply reaffirming the recommendations of the first; but without the least provision for carrying them into practical operation.

This not only confirmed the truth of the preamble referred to, but it was the completion of an unsuccessful series of efforts on the part of the Association to accomplish the great leading object of the organization, namely, the elevation of the standard of education in our profession. The Association has now a historical record of twenty-five years. A few of its founders saw clearly from the beginning that the great barrier in the way of substantial progress was the union of the teaching and licensing exclusively in the hand of an ever-varying number of colleges, necessarily engaged in active rivalry or competition with each other as to the number of students they should teach and license or graduate. But their views were regarded as too radical, and the association busied itself during the first twenty years (so far as this subject is concerned) in annually listening to reports from standing committees on medical education, and in passing resolutions and recommendations for medical colleges to execute.

Many of the reports contained criticisms upon the schools unnecessarily severe, and many of the recommendations related to details of college organizations, concerning the propriety of which each college is its own best judge. But both alike were practically unheeded by the schools. At the meeting of the Association in 1866, apparently wearied with the monotony of its own action through the twenty previous years, it dropped all further attempts to dictate to the colleges on this subject, and sent out an urgent request that those institutions would send delegates to a convention of their own, and make such revision of the system of medical education as they might deem best. It was in response to this invitation that the Medical College Convention of 1867 was held in Cincinnati, and, as a sequel, the second convention in Washington in 1870. As is well known, both were failures, so far as direct practical results from concert of action are concerned ; but, nevertheless, both were of great value indirectly —first, by the full confession contained in their proceedings that the present system of medical college organization and instruction is extremely defective, and the promulgation of some very valuable suggestions for its improvement; and, second, by the complete demonstration of the fact that the profession had no longer anything to hope from further efforts to procure practical concert of action on the part of the colleges.

It might be thought paradoxical that we claim this latter result as one of value; but it must be acknowledged that when the minds of a large body of men, like the members of the medical profession of America, are to be directed toward the accomplishment of some object of primary importance, the more clearly the impracticable methods of reaching that object are ascertained, the more speedily will they be concentrated on the method that is practicable. Viewing the past history of the Association in this aspect, we cannot agree with those who regard its efforts to advance the cause of medical education as useless. On the contrary, it has shown by abundant trial that neither reports, however ably written, nor resolves often repeated, will accomplish the desired end.

It has shown with equal force that practical concert of action among thirty or forty medical colleges, dispersed over two-thirds of a continent, and liable at all times to have their number increased by any half dozen members of the profession who should choose to organize themselves into a medical college faculty, is impossible. These results have greatly narrowed the field of future operations, and have prepared the way for concentrating the minds of the whole profession on the only feasible route to the *goal* at which they aim. That *goal* is, a higher standard of education, and greater usefulness of the profession; and the route leading to it is indicated by the first, second, and fourth resolutions adopted in 1869.

The first announces the great fundamental truth, "that whatever is done to establish and maintain a just and fair standard of medical education, must be done by the profession itself, through its own voluntary organizations. It must cease calling on *Hercules*, and put its own shoulder to the wheel, if it would see the car of progress move onward and upward in its proper course.

To invite the interference of legislative bodies, either State or National, is to place our interests in the hands of a power wholly incompetent to comprehend either our wants or the wants of the community at large—a power which, in medical matters, has thus far proved as unstable as the sands on the beach, being swayed by every popular breeze of quackery, and touching our interests only to embarrass them. Neither of the other learned professions ask for the interposition of legislation. The one regulates the qualifications and conduct of its members through its organized dioceses, synods, conferences, etc.; and the other through the examinations of the judges of the various courts. And it only needs a more perfect organization to enable our profession to exercise an efficient control over everything pertaining to its honor and usefulness.

To appeal to the medical colleges further, after the experience of the last twenty years, would be folly. An ever-varying number of institutions, each invested with certain chartered rights, subject to constant changes and necessary rivalries, cannot, in the nature of things, establish and maintain the concert of action which is necessary to accomplish the objects so much needed by our profession. The fact that a large proportion of medical students do not enter a medical college until they have passed one-third or one-half of their period of study, and then often change from one college to another in their progress, renders it impracticable for such institutions either to regulate the preliminary education of students, or to preserve any uniform standard of medical acquirements.

Medical colleges should be regarded simply as institutions for imparting instruction in all the departments of medical science and art. Restrict them to this one grand object, and all their rivalries would tend directly to elevate their character and enlarge the sphere of their usefulness. It is only when they go beyond this one noble purpose, and grant degrees that are regarded as a license to practice medicine and surgery, that they admit an element into their rivalry that necessarily tends downwards. Place the colleges in a position where the student, in choosing which he shall attend, has the sole question before him, *where can I gain the greatest amount of medical knowledge for a given sacrifice of time and money?* and every impulse of his nature impels him towards the school whose curriculum is most complete, whose facilities for demonstration and clinical instruction are the most ample, and the members of whose faculty have attained the highest reputation. And this would necessarily compel the schools to make their rivalry or competition in the same direction. Bnt leave the colleges in a position where the main question in the mind of the student, when choosing which he will attend, is, where can I gain the diploma which will admit me into the ranks of the profession *in the shortest time, and with the least expenditure of money?* and you just as certainly retain in them an element that strongly tends to keep the college curriculum, the term of lectures, and the standard of acquirements, as low as is possible without losing even the semblance of respectability.

These positions are founded on laws of social science and mental philosophy, as fixed in their operation as the laws that govern the physical world. And they are abundantly confirmed by the history of the profession, both in Europe and America.

They point with the clearness of mathematical demonstration to the truth already announced, that the profession itself, in its associated capacity, alone possesses the power and sustains the responsibility of regulating and enforcing the general standard of education for all of its members.

And the sooner this Association, as the representative of the whole profession, clearly recognizes its own responsibility, and, instead of continuing vain attempts to shift that responsibility upon medical colleges by reiterating resolutions about the length of lecture terms, college fees, etc., brings the combined influence of its wisdom and power to bear upon the State and Local societies for the accomplishment of the three following objects, the sooner will it accomplish the great leading object of its own organization. These objects are: First, to agree upon a fair, reasonable standard of education to be exacted of the student before he is permitted to commence his medical studies. Second, to define what shall constitute a fair standard of medical education to be required as a condition of admission into the ranks of the profession. Third, to procure the establishment of suitable Tribunals or Boards of Examiners in each State, for practically enforcing both the standard of preliminary and of medical education. In the prosecution of this work, by which the medical colleges would become restricted to their legitimate sphere of teaching, great care should be taken to have the regulations of the Examining boards such, that each medical student would find it necessary to spend a fair share of his period of study in such schools as possessed all the advantages necessary for imparting adequate instruction in every department of medical knowledge.

By doing this, we should indirectly do more to elevate the character of our medical colleges, than all the direct attempts to interfere with them have accomplished during the last twenty years. A careful examination of the resolutions adopted in 1869, and read in the beginning of this report, will show that they provide for all the objects here set forth, except that relating to preliminary education. And yet, this is, perhaps, of more practical importance than any other. It has long been the somewhat sarcastic reply of those connected with the medical colleges, when accused of graduating incompetent students, that the schools do the best they can with the *material* sent to them from the offices of private preceptors. And from many years of observation, both as practitioners and teachers, we are fully satisfied that if some method could be devised by which every young man entering upon the study of medicine should be required to have a respectable general education, it would speedily result in a marked elevation of the educational character and usefulness of the whole profession. Deeply impressed with the importance of the views expressed in this report, your Committee recommend the adoption of the following resolutions in addition to those adopted in 1869:

*Resolved*, That each State and Local Medical Society be requested to provide, as a permanent part of its organization, a Board of Censors for determining the educational qualifications of such young men as propose to commence the study of medicine, and that no member of such societies be permitted to receive a student into his office until such student presents a certificate of proper preliminary education from the Censors appointed for that purpose or a degree from some literary college of known good standing.

*Resolved*, That a more complete organization of the profession in each State is greatly needed for the purpose of affording a more efficient basis both for educational and scientific progress.

*Resolved*, That a committee of three be appointed for the purpose of continuing the correspondence with the State Medical Societies, and of asking their earnest attention to the foregoing resolutions in addition to those submitted for their action iu 1869.

All of which is respectfully submitted.

N. S. DAVIS,
J. M. TONER,
J. S. WEATHERLY.

